# Brown Carbon Emissions from Biomass Burning under Simulated Wildfire and Prescribed-Fire Conditions

**DOI:** 10.1021/acsestair.4c00089

**Published:** 2024-08-21

**Authors:** Chase K. Glenn, Omar El Hajj, Zachary McQueen, Ryan P. Poland, Robert Penland, Elijah T. Roberts, Jonathan H. Choi, Bin Bai, Nara Shin, Anita Anosike, Kruthika V. Kumar, Muhammad Isa Abdurrahman, Pengfei Liu, I. Jonathan Amster, Geoffrey D. Smith, Steven Flanagan, Mac A. Callaham, Eva L. Loudermilk, Joseph J. O’Brien, Rawad Saleh

**Affiliations:** † School of Environmental, Civil, Agricultural, and Mechanical Engineering, University of Georgia, Athens, Georgia 30602, United States; ‡ Department of Chemistry, University of Georgia, Athens, Georgia 30602, United States; § School of Earth and Atmospheric Sciences, Georgia Institute of Technology, Atlanta, Georgia 30332, United States; ∥ USDA Forest Service Southern Research Station, Athens, Georgia 30602, United States

**Keywords:** wildland fire, smoke, combustion conditions, fire radiative energy, organic aerosol, chromophores, light absorption

## Abstract

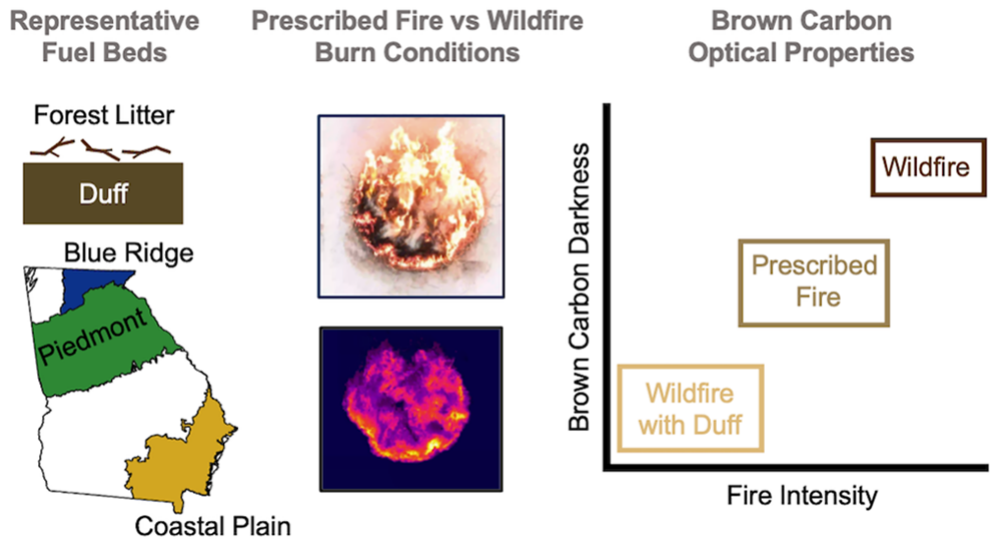

We investigated the light-absorption properties of brown carbon (BrC) as part of the Georgia Wildland-Fire Simulation Experiment. We constructed fuel beds representative of three ecoregions in the Southeastern U.S. and varied the fuel-bed moisture content to simulate either prescribed fires or drought-induced wildfires. Based on decreasing fire radiative energy normalized by fuel-bed mass loading (FRE_norm_), the combustion conditions were grouped into wildfire (Wild), prescribed fire (Rx), and wildfire involving duff ignition (WildDuff). The emitted BrC ranged from weakly absorbing (WildDuff) to moderately absorbing (Rx and Wild) with the imaginary part of the refractive index (*k*) values that were well-correlated with FRE_norm_. We apportioned the BrC into water-soluble (WSBrC) and water-insoluble (WIBrC). Approximately half of the WSBrC molecules detected using electrospray-ionization mass spectrometry were potential chromophores. Nevertheless, *k* of WSBrC was an order of magnitude smaller than *k* of WIBrC. Furthermore, *k* of WIBrC was well-correlated with FRE_norm_ while *k* of WSBrC was not, suggesting different formation pathways between WIBrC and WSBrC. Overall, the results signify the importance of combustion conditions in determining BrC light-absorption properties and indicate that variables in wildland fires, such as moisture content and fuel-bed composition, impact BrC light-absorption properties to the extent that they influence combustion conditions.

## Abstract 2

This study reports that the difference in intensity between prescribed fires and drought-induced wildfires leads to differences in optical properties of light-absorbing organic aerosol in the emissions.

## 1 Introduction

Wildland fires are important for maintaining forest ecological health and development.^1^ They encompass wildfires, which are ignited unintentionally, and prescribed fires, which are ignited intentionally for the purpose of forest management.^2^ In the U.S., the frequency, intensity, and size of wildland fires were historically controlled by prescribed fires,^3^ but the trend has shifted in recent decades. On average in the U.S., prescribed fires (mostly in the Southeastern U.S.) and wildfires (mostly in the Western U.S.) currently cover similar burned areas annually of ∼3 million ha each.^4,5^ However, wildfires exhibit significant year-to-year variability and have been increasing in frequency due to prolonged heatwaves and droughts.^6−9^

While the general view of wildfires may be skewed toward high-severity crown fires that consume the tree canopy, most wildfires occur at low and moderate severities.^10^ These fires primarily consume surface fuels, typically comprised of forest litter that accumulates on top of the forest floor. Wildfires can take place at widely different atmospheric conditions, but the majority are drought-induced and thus feature dry fuel beds. Prescribed fires, however, are carried out during favorable atmospheric conditions, often referred to as a ‘prescription window,’ where the fuel bed is neither too dry nor too moist.^2^ The differences in fuel-bed moisture content between prescribed fires and drought-induced wildfires are expected to lead to differences in combustion conditions and consequently, differences in smoke emissions. These differences are further exacerbated for forest floors that contain duff, a layer of partially decomposed forest litter that accumulates over decades in unburned forests.^11^ Prescribed fires are designed to avoid the ignition of duff, but duff can become available for combustion in drought-induced wildfires leading to drastically different combustion conditions and smoke production regimes than those associated with surface fuels.^12,13^ Given the current debate regarding the utility of prescribed fires as effective tools for wildland management,^2,4^ it is important to characterize the differences between wildfire and prescribed-fire emissions to enable quantifying their effect on air quality and atmospheric radiative balance in order to inform relevant policies.

The Georgia Wildland-fire Simulation Experiment (G-WISE) involved a systematic investigation of the differences in smoke emissions between fuel beds conditioned at moisture contents representative of prescribed fires and drought-induced wildfires. The experiments included fuel beds that contained surface fuels only as well as fuel beds that contained a duff layer underneath the surface fuels. This paper presents results from G-WISE focused on the emissions of light-absorbing organic aerosol, or brown carbon (BrC).^14^ Though less efficiently light-absorbing than black carbon (BC), BrC is typically emitted at substantially higher levels than BC in wildland fires and is thus an important contributor to absorption of solar radiation in the atmosphere.^15^ Accounting for BrC absorption in emissions from wildland fires was shown to improve the agreement between radiative-transfer calculations and remote-sensing observations.^16^ However, estimates of the global direct radiative effect of BrC absorption exhibit a wide range (+0.03 W m^–2^ to +0.57 W m^–2^).^17−22^ This is in part due to the poorly characterized light-absorption properties of BrC, quantified using the mass absorption cross-section (MAC) or the imaginary part of the refractive index (k).^15^ There is an abundance of studies that retrieved MAC and/or k of BrC both in field measurements^23−29^ and laboratory experiments,^30−37^ with reported values varying over several orders of magnitude.^15^ At least in part, the large variability in reported BrC light-absorption properties is due to differences in combustion conditions.^33,37^ Furthermore, BrC is comprised of molecules with highly diverse molecular structures^38^ that exhibit varying levels of solubility in water and organic solvents, with the insoluble fraction being more absorbing.^32,39,40^ Therefore, techniques that rely on solvent-extraction underestimate BrC light absorption,^32^ which is also partly responsible for the large variability in reported BrC light-absorption properties.

In this study, we investigate how the differences in combustion conditions between prescribed fires and drought-induced wildfires, which arise from differences in fuel moisture content and the availability of duff for combustion, affect BrC light-absorption properties. Furthermore, we apportion the BrC into water-soluble and water-insoluble fractions to assess the implications of relying on water extraction for retrieving the light-absorption properties.

## 2 Methods

This study was performed as part of the Georgia Wildland-fire Simulation Experiment (G-WISE). We first provide a general description of G-WISE and then focus on the analyses specific to this study.

### 2.1 Burn Experiments

#### 2.1.1 Collection of Fuel Samples and Fuel-Bed Preparation

**Figure 1:**
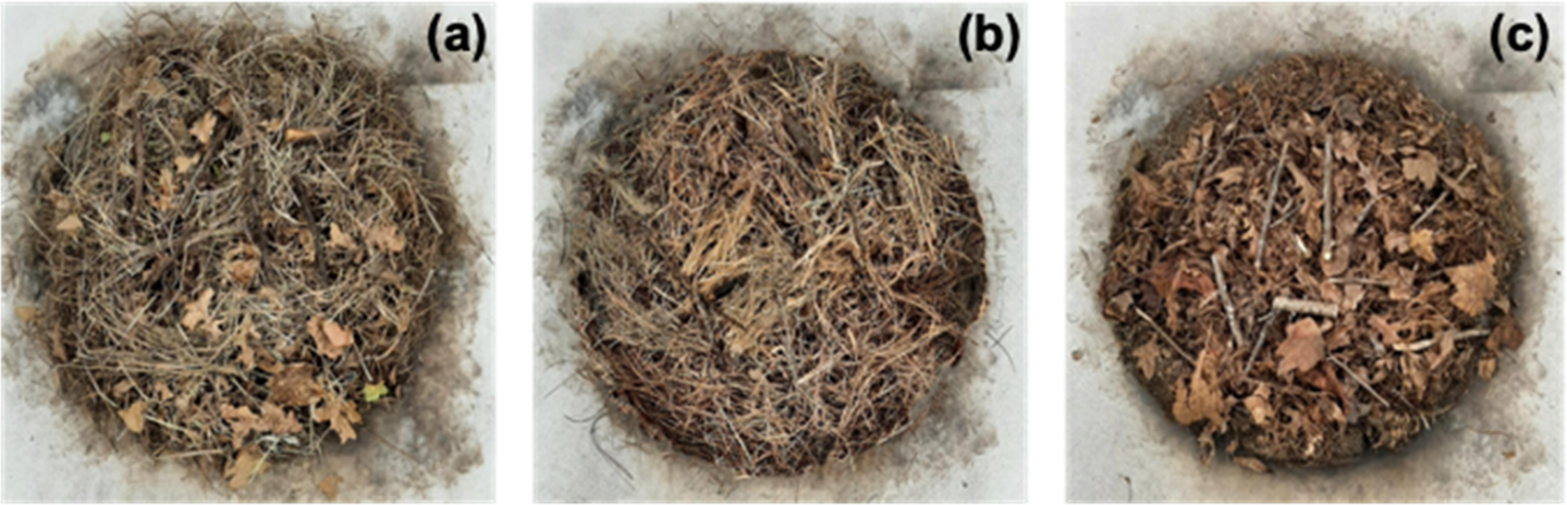
Pictures of 0.5 m^2^ fuel beds reconstructed using samples collected from (a) Piedmont, (b) Coastal Plain, and (c) Blue Ridge.

G-WISE was an intensive laboratory campaign conducted in October-November 2022 at the U.S. Forest Service Southern Research Station Prescribed Fire Science Laboratory on the campus of the University of Georgia in Athens, GA. G-WISE involved performing burn experiments of fuel beds constructed using samples collected from 3 ecoregions in Georgia: Oconee National Forest (Piedmont), Fort Stewart (Coastal Plain), and the Chattahoochee National Forest in the southern Blue Ridge mountains (Blue Ridge). These ecoregions are representative of the Southeastern U.S. forests.^41^ The Piedmont and Coastal Plain fuel beds featured surface fuels, which included fine fuels (needles, leaves, litter) as well as woody fuels. The Blue Ridge fuel beds also included a duff layer underneath the surface fuels. Importantly, the experiments strived to simulate similar combustion conditions as would be encountered in the field by maintaining two aspects. First, the fuel beds recreated the loadings (kg m^–2^), proportions (fine fuels, woody fuels, duff), and 3D structures of the fuel beds observed in the field using extensive sampling as well as light detection and ranging (LIDAR) measurements.^42^ Second, we employed a fuel-bed area of 0.5 m^2^, which corresponds to the scale of a “wildland fuel cell” unit, based on field observations.^43^ Specifically, Hiers et al.^43^ demonstrated that beyond the 0.5 m^2^ scale, fire behavior becomes spatially independent. Therefore, employing a fuel-bed area of 0.5 m^2^ captures the small-scale interdependence of fire behavior and consequently smoke production encountered in the field. Figure 1 shows representative fuel beds that were constructed during G-WISE for the 3 ecoregions.

The moisture content of the fuel beds was conditioned to two levels, which are representative of either prescribed fires or drought-induced wildfires. For prescribed-fire conditions, the fine fuels and woody fuels were conditioned to moisture contents of 10%–11% and 32%–50%, respectively, which are close to the midpoint of the prescription window usually employed in these ecoregions.^2^ For the fuel beds that included duff (Blue Ridge), the duff layer was used as collected from the field and had a moisture content of approximately 50%. For the drought-induced wildfire conditions, the fuel beds were conditioned to below 4% moisture content. While bearing in mind that wildfires can occur at any moisture content, the majority of the burned areas consumed by wildfires occur under drought (dry) conditions.^2^ For the purpose of the discussion in this paper, we will drop the “drought-induced” qualifier in the subsequent sections. Further details on fuel proportions, mass loadings, and moisture content are given in Table S1 in the Supporting Information (SI).

The experiments involved 6 experimental permutations based on the combination of ecoregion (Piedmont (P), Coastal Plain (CP), Blue Ridge (BR)) and moisture content (wildfire (Wild) or prescribed fire (Rx)): P-Wild, P-Rx, CP-Wild, CP-Rx, BR-Wild, and BR-Rx. Each permutation was repeated 3 times.

#### 2.1.2 Experimental Procedure

**Figure 2:**
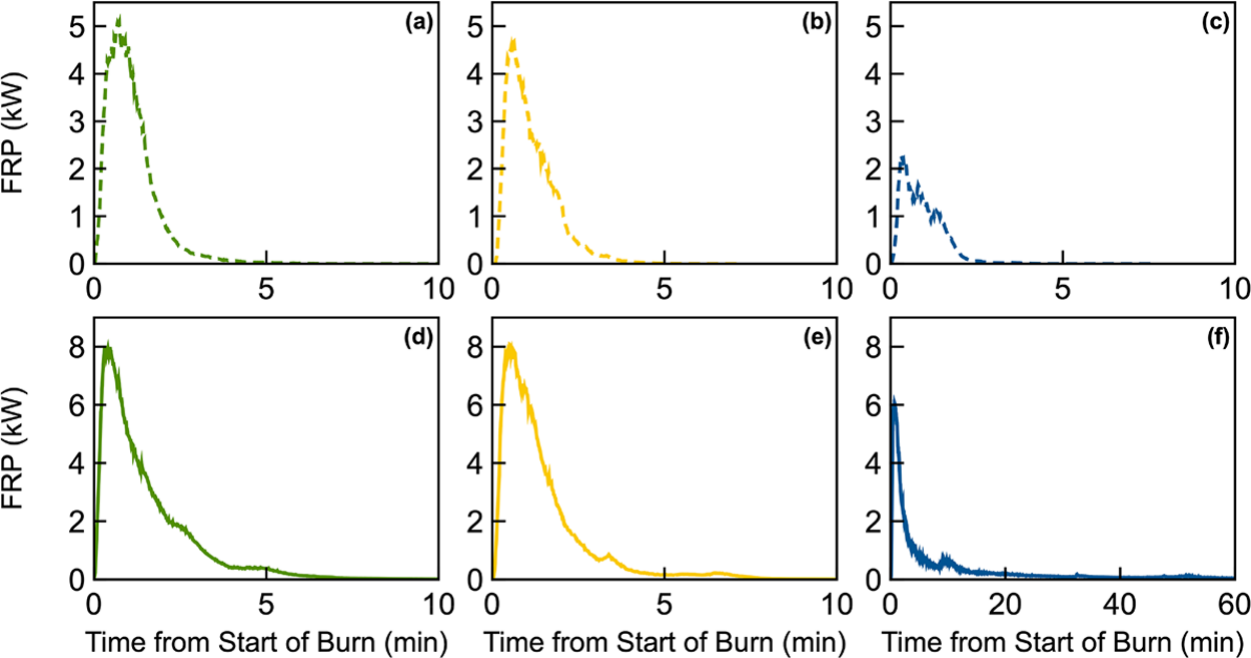
Time series of fuel-bed fire radiative power (FRP) of representative burns for (a) P-Rx (10/31/2022), (b) CP-Rx (11/06/2022), (c) BR-Rx (11/11/2022), (d) P-Wild (10/25/2022), (e) CP-Wild (11/02/2022), and (f) BR-Wild (11/12/2022).

The burns were conducted in a 1000 m^3^ burn room equipped with an array of fans that were used to attain well-mixed conditions. Sampling lines were extended from the burn room to an adjacent instrument room in order to perform both online measurements as well as collect filter samples for offline analyses. The fuel bed was placed on top of a scale to monitor fuel consumption in real-time. We monitored the fire behavior at 30 Hz using a radiometric thermal imager (Flir A655 sc), which was down-sampled to 1 Hz thermography to retrieve real-time combustion temperatures and calculate the fire radiative power (FRP) throughout the burn as elaborated below. The burns typically concluded within 10 min (Figure 2), as inferred from the real-time temperatures retrieved from the infrared camera measurements falling below 573 K within all pixels. A notable exception was for experiments that involved duff ignition, where the burn would carry on at low temperatures (FRP) for approximately 60 min.

The smoke reached well-mixed conditions in the burn room within 10 min of the conclusion of the burn, as inferred from the aerosol volume concentrations obtained from integrating size distribution measurements performed using a scanning mobility particle sizer (SMPS, TSI, 3082) reaching a peak level, and then dropping with an e-folding time scale of approximately 4 h due to particle wall-losses and infiltration of ambient air into the burn room. After reaching well-mixed conditions, we collected filter samples for various offline analyses for a period of 30–60 min. The relatively high smoke concentrations were advantageous for minimizing the filter sampling time but were too high for online aerosol and gas-phase measurements. Therefore, after filter collection was completed, we vented the smoke from the burn room by bringing in fresh ambient air via a ventilation system until the aerosol volume concentration reached approximately 200–300 μm^3^ cm^–3^, after which online measurements commenced.

#### 2.1.3 Measurements Used in this Study

G-WISE involved the deployment of extensive online and offline smoke characterization techniques. Here, we list the techniques that were utilized in the analyses that pertain to this study. The major goal of this paper is to assess the dependence of BrC light-absorption properties on fuel-bed composition and moisture content (prescribed fire versus wildfire). In addition to categorizing the burns into the 6 permutations listed in section 2.1.1, we also characterized the combustion conditions using online fuel-consumption measurements and FRP measurements, as detailed in section 2.2. We retrieved the light-absorption properties of the BrC aerosol, as well as the water-soluble BrC (WSBrC) and water-insoluble BrC (WIBrC). To that end, we utilized online measurements of aerosol absorption coefficients (*b*_abs_, Mm^–1^) at 3 wavelengths (406, 532, and 660 nm) using a photoacoustic spectrometer (Multi-PAS)^44^ and size distributions over the range of 16–1000 nm using an SMPS. We also utilized offline thermal-optical measurements of the elemental carbon (EC) and organic carbon (OC) fractions of the aerosol using an OCEC analyzer (Sunset Laboratory Inc., Model 5 L) as well as light-absorption measurements of WSBrC using UV–vis spectroscopy. The details of these analyses and their utility to retrieve the light-absorption properties of BrC aerosol, WSBrC, and WI-BrC are described in sections 2.3–2.5. Finally, we characterized the chemical composition of the WSBrC using electrospray ionization Fourier-transform ion cyclotron resonance mass spectrometry (ESI-FTICR-MS), as detailed in section 2.6.

### 2.2 Fire Radiative Power and Fire Radiative Energy

For each burn, we calculated the fuel-bed fire radiative power (FRP, W) at 1-s resolution using temperatures retrieved from the radiometric thermal imager assuming gray-body radiation and using a minimum threshold of 573 K:^45^
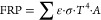
1where T is the temperature (K), ε is the emissivity (assumed to be 0.98),^45^ σ = 5.67 × 10^–8^ W m^–2^ K^–4^ is the Stefan–Boltzmann constant, and A is pixel area.

Figure 2 shows representative time series of FRP over the course of a burn for the 6 experimental permutations. We integrated FRP over the duration of the burn to obtain the fire radiative energy (FRE, MJ) and normalized it by the available fuel mass loading to obtain FRE_norm_ (MJ kg^–1^) for each burn. Whereas FRE is the total amount of radiative energy released from a fuel bed and is dependent on the fuel mass loading,^46−49^ FRE_norm_ is a measure of how efficiently the fuel is converted to radiative energy and is therefore an indirect measure of combustion efficiency, which we use to characterize combustion conditions. We note that the duff layer in the Blue Ridge fuel beds was not available for combustion (i.e., did not ignite) under prescribed-fire conditions, thus only the surface fuel mass was used to calculate FRE_norm_ for BR-Rx. For the rest of the experimental permutations, all the fuel bed was available for combustion, thus the total fuel mass loading was used in the FRE_norm_ calculations.

### 2.3 Light-Absorption Properties of Brown Carbon Aerosol

We utilized a combination of online and offline measurements and optical closure (Mie theory) calculations to retrieve the wavelength-dependent imaginary part of the refractive index (k) of the BrC aerosol.^33,37,50,51^ The wavelength-dependent k can be represented using a power-law functional dependence on wavelength:
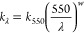
2where k_λ_ is k at any wavelength, *k*_550_ is k at 550 nm, and w is the wavelength dependence.^15^

Therefore, k_λ_ can be represented using two parameters, namely *k*_550_ and w, which were retrieved from optical closure by fitting Mie theory calculations to b_abs_ measurements (at 406, 532, and 660 nm) using the Multi-PAS. The absorption coefficient of BrC was obtained from the measurements as
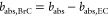
3where b_abs_ is the total measured absorption coefficient that includes contribution from BrC and EC, and b_abs,EC_ is the EC absorption coefficient calculated using Mie theory assuming externally mixed EC and BrC particles.

Calculating b_abs,EC_ requires information on the EC complex refractive index and size distribution. We used EC complex refractive index of m = 1.85 + 0.71*i*.^52^ We assumed that the EC size distribution had the same shape as the overall aerosol size distribution measured using the SMPS and was scaled based on the relative abundance of EC and organic matter (OM) in the aerosol. The EC and OM mass concentrations were obtained from thermal-optical measurements using the OCEC analyzer following the same procedure in Atwi et al.^32^ and Glenn et al.^53^ Both quartz (Q) and quartz behind Teflon (QBT) filters were analyzed in the OCEC analyzer using the Niosh-870 protocol (see SI Table S2).^54^ EC was determined directly from the Q filter measurements. The OC measurements were corrected for vapors adsorbed on the Q filter as^55^
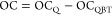
4where OC_Q_ and OC_QBT_ correspond to the OC measured on the Q and QBT filters, respectively. EC and OC fractions from all experiments are given in SI Table S3. OM was calculated by converting OC to an organic-mass basis assuming OM/OC of 1.8.^56−58^ We note that the relative abundance of OM and EC was calculated based on filter samples collected prior to diluting the smoke in the burn room while the optical-closure calculations were performed based on SMPS and Multi-PAS measurements after dilution (section 2.1.2). Some semivolatile organic compounds could potentially partition from the particle phase to the gas phase upon dilution, which could lead to overestimating OM concentrations and underestimating k_λ_ retrieved from the optical-closure analysis.

The retrieval process of BrC light-absorption properties described above is based on the assumption that the EC and BrC particles are spherical and externally mixed, which does not represent their true morphology and mixing state^59,60^ and thus impacts the retrieved light-absorption properties.^61,62^ However, Saleh et al.^62^ showed that for use in chemical-transport and climate models, it is recommended that the assumed morphology and mixing state in retrievals of BrC light-absorption properties be consistent with those employed in the models. Radiative-transfer calculations in regional and global models are typically based on Mie theory (i.e., assume spherical particles) and have employed both internal-mixing^17,22,63^ and external-mixing^16,18,20,64,65^ assumptions. Therefore, if the light-absorption properties retrieved from this study are to be used in radiative-transfer calculations, we recommend employing external-mixing assumption in the model.

### 2.4 Light-Absorption Properties of Water-Soluble Brown Carbon

We retrieved the imaginary part of the refractive index of WSBrC (k_WSBrC_) using offline UV–vis spectroscopy following a procedure similar to Atwi et al.^32^ and Cheng et al.^66^ First, we performed passive extraction (i.e., without sonication) of both Q and QBT filters in 5 mL of ultrapure water at room temperature for 24 h. This method is effective at removing water-soluble OC (i.e., WSBrC) from the filter without forcibly dislodging water-insoluble OC (i.e., WIBrC).^40,67^ We then filtered the water extracts through a glass syringe with a metal luer-lock tip loaded with a 13 mm PTFE filter (0.2 μm, Sterlitech Corporation, PTU021350) to remove any residual insoluble material. We measured the absorbance of the extracts of both Q and QBT filters using a UV–vis Spectrometer (Agilent, Cary 60) over the range of 200 nm −800 nm at a 1 nm resolution. The absorbance corrected for adsorbed vapors (A(λ)_WSBrC_) was obtained as
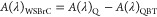
5where A(λ)_Q_ and A(λ)_QBT_ are the absorbance measurements of the extracts of the Q and QBT filters, respectively.

We used A(λ)_WSBrC_ to calculate the absorption coefficient (α_*WSBrC*_, cm^–1^) and subsequently k_WSBrC_:^32^
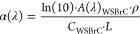
6
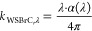
7where ρ (1.2 g cm^–3^) is the assumed density of the extracts,^32^*L* (1 cm) is the optical path length, and *C*_WSBrC_ is the mass concentration of WSBrC in the solution obtained as
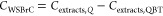
8where C_extracts,Q_ and C_extracts,QBT_ are the concentrations of the extracts of Q and QBT filters, respectively, which were determined as follows.

We pipetted 200 μL of the corresponding solutions onto a prebaked punch from a Q filter. The punch was dried under a stream of clean, dry air at a flow rate of 10 LPM for 30 min and the OC mass on the punch was determined using the OCEC analyzer (by running NIOSH-870 protocol). As before, we converted OC to OM assuming OM/OC of 1.8. The details of these calculations and the associated uncertainties are given in the SI.

### 2.5 Light-Absorption Properties of Water-Insoluble Brown Carbon

To retrieve the imaginary part of the refractive index of WIBrC (k_WIBrC_), we assumed that WSBrC and WIBrC were well-mixed and that k_BrC,aerosol_ is a volume-weighted average of k_WSBrC_ and k_WIBrC_. Then, k_WIBrC_ can be calculated as^32^
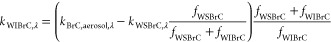
9where k_BrC,aerosol_ is obtained from the optical closure analysis (section 2.3), k_WSBrC_ is obtained from offline UV–vis measurements (section 2.4), and f_WSBrC_ and f_WIBrC_ are the fractions of WSBrC and WIBrC, respectively.

The procedure to obtain f_WSBrC_ and f_WIBrC_ was as follows. Punches from both the Q and QBT filters were analyzed in the OCEC analyzer to obtain OC_Q_ and OC_QBT_ as described in section 2.3. Separate punches from both the Q and QBT filters underwent passive extraction in 3 mL of ultrapure water for 24 h. After extraction, each punch was dried under a stream of clean, dry air at a flow rate of 10 LPM for 30 min. The samples then underwent OCEC analysis which yielded OC_Q,WI_ and OC_QBT,WI_. We then calculated OC_WS_ and OC_WI_ as
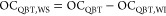
10
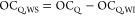
11

Then OC_WS_ was obtained from equation 10 and equation 11 as
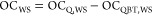
12and OC_WI_ was obtained as
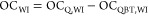
13

Similar to the procedure described in Section 2.4, OC_WS_ and OC_WI_ were converted to organic-mass basis (OM_WS_ and OM_WI_) assuming OM/OC of 1.8. Previous work demonstrated that assuming an OM/OC of 1.5–2 did not significantly affect the retrieval of light-absorption properties.^32^ The total carbonaceous mass (TM) was obtained as
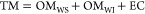
14where EC was obtained directly from the OCEC analysis of the unextracted Q punch. The fractions of WSBrC, WIBrC, and EC were then obtained as
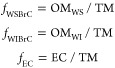
15

We note that the WSBrC and WIBrC fractions are operationally defined. However, based on the sensitivity test detailed in the SI, doubling the extraction time and the extraction volume had negligible effect on the measured WSBrC and WIBrC fractions. This indicates that under our experimental conditions, there were neither kinetic limitations nor solubility limitations associated with the extraction process. Therefore, the WSBrC and WIBrC fractions reported in this study, though operationally defined, can be practically generalized.

### 2.6 Chemical Speciation of Water-Soluble BrC

The water extracts were analyzed using electrospray ionization Fourier-transform ion cyclotron resonance mass spectrometry (ESI-FTICR-MS). ESI is widely used in chemical composition analysis of biomass-burning OA^53,68−74^ because the biomass-burning OA molecules include functional groups that are efficiently ionized by ESI.^38,75^ Analysis was performed in negative ionization mode on a Bruker SolariX XR 12 T FTICR mass spectrometer over a *m*/*z* range of 70–1000. The transient length was 1.667 s which gave a mass resolution of ∼430,000 at *m*/*z* 400. The capillary was set to 4500 V with an end plate offset of −800 V. The dry gas rate was 4.0 L/min, nebulizer gas pressure was 0.8 bar, and the dry temperature was maintained at 200 °C. Spectra for each sample were acquired in triplicate, and each spectrum was an average of 48 scans.

We prepared a blank solution by extracting a clean filter using the same procedure described in section 2.4. Each spectrum was blank subtracted in Bruker Data Analysis using the Xpose method. The blank-subtracted mass spectra were then analyzed using MFassignR^76^ to obtain molecular assignments. Peaks first underwent carbon, hydrogen, and oxygen (CHO) assignments using an initial mass tolerance of 1 ppm. Then ^13^C and ^34^S isotopes were identified and filtered so that only monoisotopic peaks were selected. The monoisotopic peaks then underwent an internal mass recalibration.^77^ The final elemental composition assignments for recalibrated peaks were obtained using a constraint that the number of nitrogen atoms is less than or equal to three. In all experiments, sulfur-containing compounds constituted less than 2% of the assignments and were thus not considered in the analysis.

## 3 Results and Discussion

### 3.1 Brown Carbon Optical Classification

**Figure 3:**
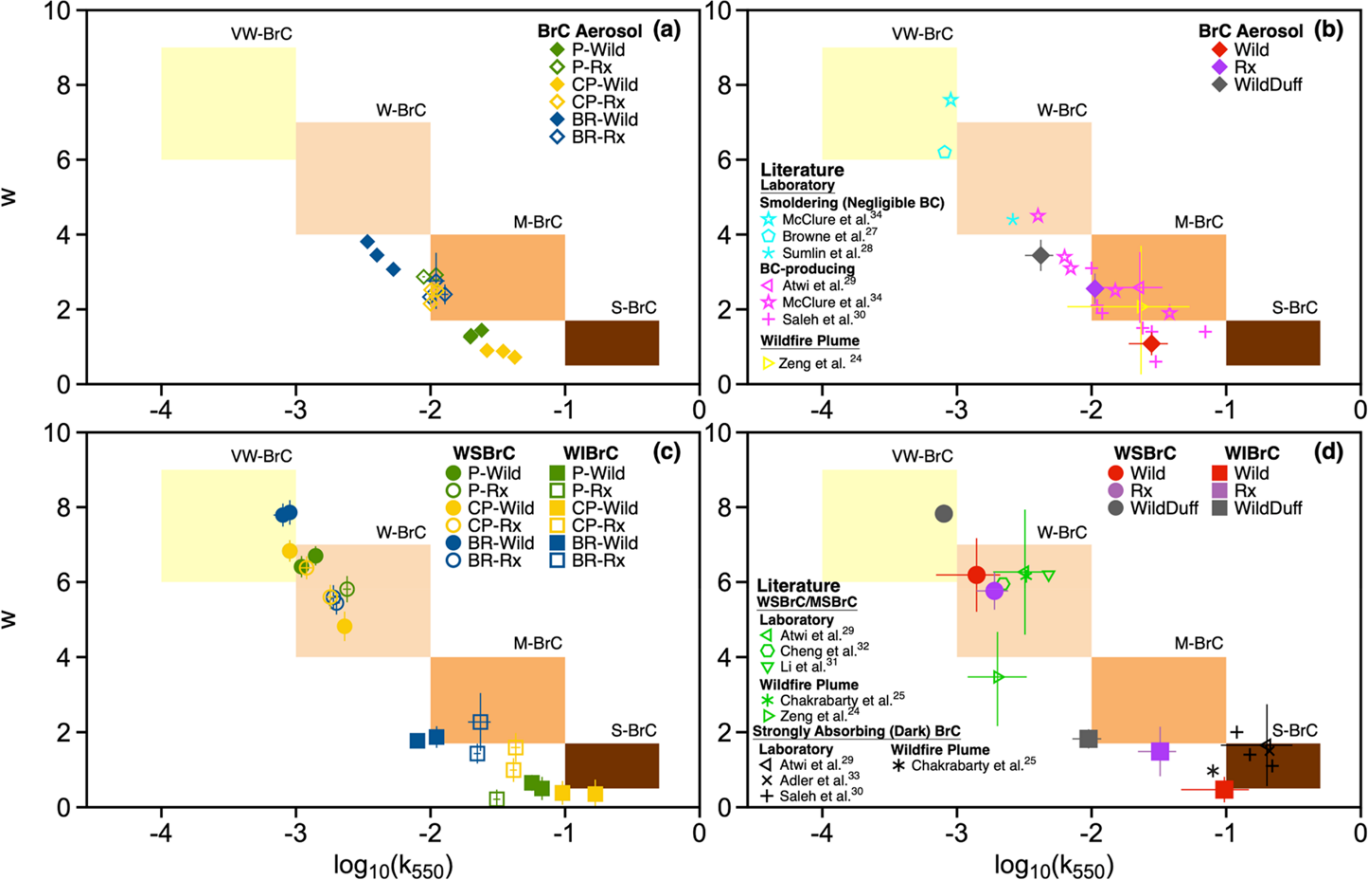
Light-absorption properties of BrC aerosol, WSBrC, and WIBrC from all experiments plotted in log_10_(*k*_550_)–*w* space. The shaded regions represent the optical classes proposed by Saleh:^15^ very weakly absorbing BrC (VW-BrC), weakly absorbing BrC (W-BrC), moderately absorbing BrC (M-BrC), and strongly absorbing BrC (S-BrC). Error bars represent uncertainty, calculated as described in the SI. Numerical values of each of the data points are given in SI Table S4. (a) BrC aerosol for each of the six experimental permutations. (b) Averages of the data points in panel (a) for the three groups: wild, Rx, and WildDuff. Also shown are values calculated from data obtained from previous studies. Numerical values of each data point and information on how *k*_550_ and *w* were calculated from each study are given in SI Table S5. (c) WSBrC and WIBrC for the six experimental permutations. (d) Averages of the data points in panel c for the three groups: wild, Rx, and WildDuff. Also shown are values calculated from data obtained from previous studies for WSBrC and methanol-soluble BrC (MSBrC) and strongly absorbing (dark) BrC. Numerical values of each data point and information on how *k*_550_ and *w* were calculated from each study are given in SI Table S5.

The light-absorption properties of BrC aerosol from all experiments are presented on log_10_(*k*_550_)-w space^15^ in Figure 3a. Also shown are the BrC optical classes proposed by Saleh,^15^ where increasing *k*_550_ and decreasing w are indicative of increasing BrC absorption (i.e., darker BrC). As evident in Figure 3a, the BrC aerosol is clustered in 3 groups with decreasing absorption: (1) Wild (including P-Wild and CP-Wild), (2) Rx (including P-Rx, CP-Rx, and BR-Rx), and (3) Wild with Duff (WildDuff; including BR-Wild). The light-absorption properties of these groups are shown in Figure 3b. This finding provides a practical first-order estimation of *k*_550_ and w of BrC emissions from wildland fires: (1) *k*_550_ = 0.028 ± 0.01 and w = 1.08 ± 0.31 for wildfires, (2) *k*_550_ = 0.011 ± 0.001 and w = 2.55 ± 0.40 for prescribed fires, and (3) *k*_550_ = 0.004 ± 0.001 and w = 3.44 ± 0.42 for wildfires that involve duff combustion.

The clustering of BrC light-absorption properties from the 6 experimental permutations (P-Wild, P-Rx, CP-Wild, CP-Rx, BR-Wild, and BR-Rx) into 3 groups (Wild, Rx, and WildDuff) signifies an interplay between fuel-bed composition (P vs CP vs BR) and moisture content (Wild vs Rx). Setting the stage for dissecting this interplay requires making two points. First, there were differences in the composition of the surface fuels between P, CP, and BR. As can be visually inferred from the fuel-bed pictures (Figure 1), P and BR had appreciable amounts of oak leaves while CP had no leaves but appreciable amounts of grasses. Second, BR was the only fuel bed that contained duff (SI Table S1). Duff did not ignite in BR-Rx because of the high moisture content, but it was available for combustion in BR-Wild and dominated the emissions due to its high mass loading compared to the surface fuels (SI Table S1). Therefore, BR-Wild was the only experimental permutation that involved duff combustion.

For the 5 experimental permutations that involved combustion of surface fuels only, moisture content (Rx vs Wild) played a more important role than fuel-bed composition in dictating BrC light-absorption properties. Specifically, the BrC in Rx was less absorbing (smaller *k*_550_ and larger w) than Wild. The reason is that the higher moisture content in Rx compared to Wild led to overall lower combustion temperature (lower FRP; Figure 2). The lower combustion temperature hinders the soot-formation process and, in concordance with the brown-black continuum,^78^ produces less-absorbing BrC. This finding is in agreement with the observation in the review by Saleh^15^ that studies involving low-temperature (smoldering) biomass combustion have typically reported less-absorbing BrC compared to studies involving high-temperature (BC-producing) biomass combustion (Figure 3b).

The same reasoning can be applied to explain why BR-Wild, the only permutation that involved duff ignition, emitted by far the least-absorbing BrC. Due to its substantially higher bulk density compared to surface fuels,^79^ duff combustion is characterized by oxygen-deprived low-temperature smoldering conditions^12,13,80^ as evidenced by the long tail of low FRP in Figure 2f. Therefore, BR-Wild emitting the least-absorbing BrC is in-line with the association between BrC light-absorption properties and combustion temperature described above.

### 3.2 Water-Soluble and Water-Insoluble Brown Carbon

The light-absorption properties (*k*_550_ and w) of WSBrC and WIBrC from all experiments are shown in Figure 3c and the averages for the 3 groups (Wild, Rx, and WildDuff) are shown in Figure 3d. We note that even though the light-absorption properties of WBrC, WIBrC, and BrC aerosol were retrieved using different methods (sections 2.3–2.5), we have previously shown that the light-absorption properties obtained from these online and offline methods are consistent.^81^ Therefore, differences in *k*_550_ and w values of WBrC, WIBrC, and BrC aerosol are attributed to true differences associated with extraction efficiency rather than differences in optical measurement techniques.

For all groups, *k*_550_ of WIBrC is more than 1 order of magnitude larger than that of WSBrC. This result is in-line with the findings of Atwi et al.,^32^ who reported a two-order-of-magnitude difference between *k*_550_ of methanol-insoluble BrC (MIBrC) and methanol-soluble BrC (MSBrC) in biomass-burning emissions. Figure 3d also shows light-absorption properties of WSBrC and MSBrC from previous studies, which mostly fall within the weakly absorbing BrC class, in agreement with our results. This further confirms that relying on water or methanol extraction severely underestimates BrC absorption.^24,32^

The light-absorption properties of WIBrC approach the strongly absorbing BrC class,^15^ further confirming the existence of highly absorbing (dark) BrC in wildland-fire emissions reported in previous laboratory^32,33,36^ and field^25^ measurements (Figure 3d). It is important to note that the strongly absorbing BrC is coemitted with other less-absorbing BrC components. Therefore, detection of the strongly absorbing BrC has typically been reported in studies that involved separating it from the less-absorbing components by relying on the association between solubility, volatility, and light-absorption properties.^15,39^ Examples include isolating the BrC fraction resistant to volatilization during electron energy-loss spectroscopy (EELS) measurements^25^ or heating in a thermodenuder,^33,36^ or isolating the methanol-insoluble^32^ or water-insoluble (this study) fractions.

**Figure 4:**
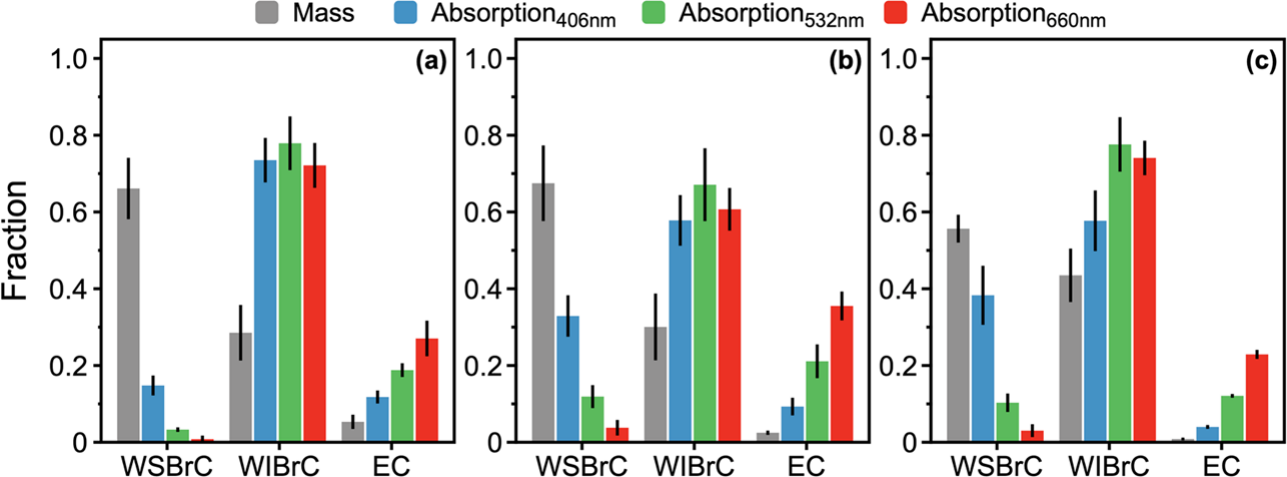
Average mass fraction of WSBrC, WIBrC, and EC and their relative contributions to absorption at 406, 532, and 660 nm for (a) Wild, (b) Rx, and (c) WildDuff. Error bars represent uncertainty, calculated as described in the SI. Numerical values of each of the data points are given in SI Table S6.

As shown in Figure 3c and 3d, the light-absorption properties of WIBrC of the 3 groups (Wild, Rx, and WildDuff) exhibit the same trend as the BrC aerosol, while those of WSBrC do not. This indicates that WIBrC is more dominant than WSBrC in dictating the BrC aerosol absorption as further illustrated in Figure 4. The mass fractions of WSBrC, WIBrC, and EC are plotted alongside their respective contribution to absorption at 406, 532, and 660 nm. Although WIBrC accounted for a substantially smaller fraction of the total carbonaceous aerosol mass compared to WSBrC, it dominated the contribution to BrC absorption at all wavelengths.

### 3.3 Chromophores in Water-Soluble Brown Carbon

**Figure 5:**
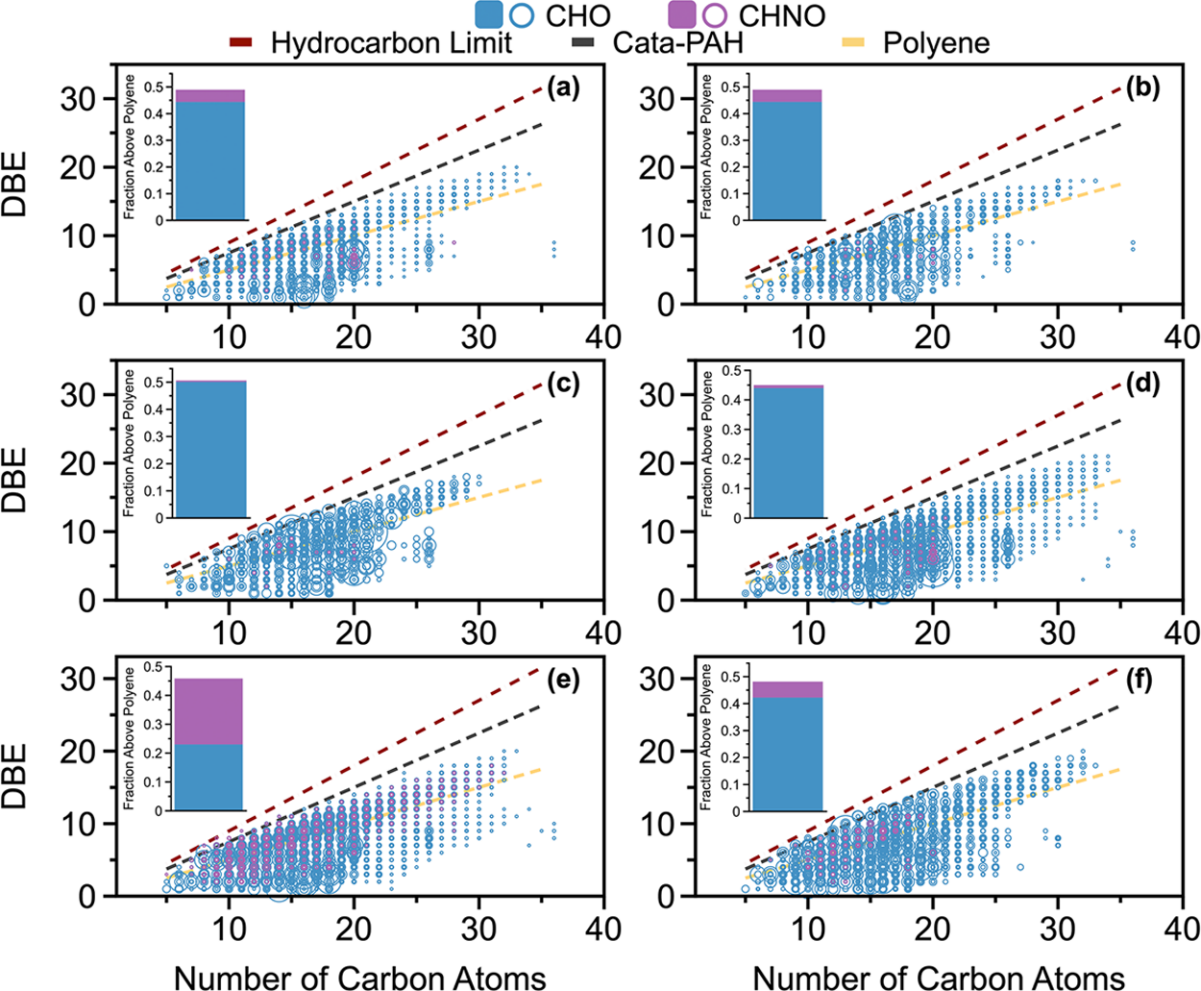
DBE versus number of carbon atoms of CHO and CHNO molecules detected by ESI-FTIRC-MS for representative burns: (a) P-Wild (915 molecular assignments), (b) P-Rx (959 molecular assignments), (c) CP-Wild (810 molecular assignments), (d) CP-Rx (1779 molecular assignments), (e) BR-Wild (2824 molecular assignments), and (f) BR-Rx (1397 molecular assignments). Symbol size is proportional to relative peak abundance. The dashed lines denote the lower bounds of polyene (DBE/C = 0.5; gold) and Cata-PAH (DBE/C = 0.75, gray), as well as the hydrocarbon limit (DBE/C = 0.9; red). The region bounded by DBE/C ≥ 0.5 and DBE/C ≤ 0.9 represents potential BrC chromophores.^72,82^ The insets represent the fraction of molecules that are potential BrC chromophores (i.e., above the polyene line).

Following the approach of Hopstock et al.,^82^Figure 5 shows double-bond equivalents (DBE) versus carbon number for WSBrC molecules detected by ESI-FTICR-MS. Based on this framework, organic molecules that fall above the polyene line are potential BrC chromophores.^72^ Consistent across all experimental permutations, approximately half of the WSBrC molecules detected by ESI-FTICR-MS fall above the polyene line. One notable distinction is the high abundance of nitrogen-containing molecules (CHNO) in BR-Wild compared to other permutations. Previous studies have shown that nitrogen-containing organic molecules in wildland-fire emissions, such as nitro-aromatics, are prominent BrC chromophores.^71,83,84^ However, BR-Wild emitted the least-absorbing BrC among all permutations (Figure 3). This seeming inconsistency with previous studies can be explained as follows. BR-Wild is the only permutation that included duff ignition and featured substantially lower combustion temperatures compared to other permutations, as further elaborated in section 3.4. Therefore, BR-Wild emissions are not expected to include significant amounts of nitro-aromatics, the formation of which take place predominantly during high-temperature flaming combustion.^83,85,86^ Duff contains elevated levels of nitrogen,^11,87^ which accumulates during the decomposition process that involves breaking down of organic nitrogen in plant litter by bacteria and fungi.^88^ Therefore, it is likely that a fraction of the nitrogen-containing molecules observed in the BR-Wild WSBrC emissions were distillation products (i.e., molecules that did not form during combustion but volatilized directly from the duff) which include functional groups that do not exhibit prominent absorption in the visible spectrum.^84^ While the absence of information on molecular structure in this study prevents confirmation, this assertion provides a plausible explanation for BrC in BR-Wild emissions being the least absorbing among all permutations.

### 3.4 Brown Carbon Light-Absorption Properties Correlated with Combustion Conditions

The results described in Section 3.1 and shown in Figure 3 indicate that BrC light-absorption properties depend on combustion conditions. Here, we explore this dependence in more detail by utilizing FRE_norm_ as a metric. As described in Section 2.2, FRE is the total radiative energy released from a burn. It has been shown to correlate with total aerosol emissions in laboratory experiments^49^ and has been utilized as a basis for developing top-down emission inventories.^89−91^ FRE depends on available fuel mass loading and is therefore not necessarily indicative of burn conditions. For example, the same FRE could be released from a low-temperature smoldering fire with high fuel mass loading and a high-temperature flaming fire with low fuel mass loading. This is clearly illustrated in our experiments, where BR-Wild was the most smoldering among all experimental permutations but had the largest FRE because of the high duff mass loading (SI Table S7).

**Figure 6:**
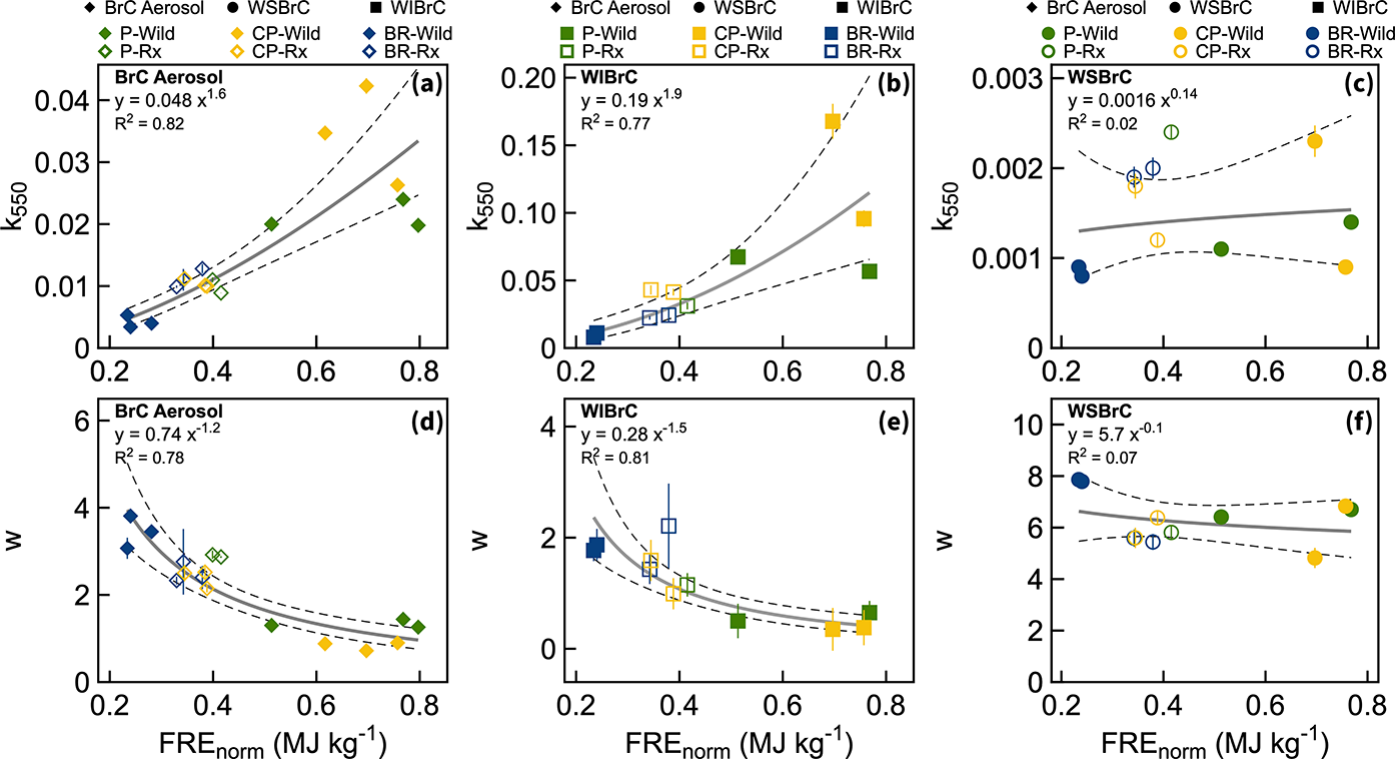
Imaginary part of the refractive index at 550 nm and wavelength dependence as a function of normalized fire radiative energy for the six experimental permutations of (a, d) BrC aerosol, (b, e) water-insoluble BrC, and (c, f) water-soluble BrC. Error bars represent uncertainties (Table S4). Solid lines are power-law fits and dashed lines represent 95% confidence bounds.

Being normalized by available fuel mass loading, FRE_norm_ can be thought of as an effective radiative heating value of the fuel bed. As shown in Figure 6, FRE_norm_ is lowest for WildDuff (BR-Wild), followed by Rx (P-Rx, CP-Rx, BR-Rx) and Wild (P-Wild, CP-Wild). These results indicate that for the five experimental permutations that involved combustion of surface fuels only (P-Wild, P-Rx, CP-Wild, CP-Rx, BR-Rx), combustion conditions were largely determined by fuel moisture content. The higher moisture content in Rx led to substantial reduction in FRE_norm_ compared to Wild because of the additional energy required to evaporate the water (enthalpy of vaporization),^92^ which was more dominant than any potential effects the differences in fuel-bed composition had on FRE_norm_. However, combustion conditions in BR-Wild were highly influenced by the oxygen-deprived low-temperature duff combustion, leading to substantially lower FRE_norm_ compared to experimental permutations that involved combustion of surface fuels only.

The BrC aerosol light-absorption properties are well-correlated with FRE_norm_. *k*_550_ increases with increasing FRE_norm_ (Figure 6a) and w decreases with increasing FRE_norm_ (Figure 6d), confirming that higher-temperature fires emit more-absorbing BrC. Based on these findings, we derived parametrizations of *k*_550_ and w as a function of FRE_norm_. Similar to the BrC aerosol, *k*_550_ and w of WIBrC are well-correlated with FRE_norm_ (Figure 6b and 6e). However, *k*_550_ and w of WSBrC exhibit no dependence on FRE_norm_ (Figure 6c and 6f). Even though this study did not allow for direct comparison between the WSBrC and WIBrC components due to lack of chemical speciation of WIBrC, the findings in Figure 6 point to a difference in the formation pathways between the chromophores represented in WIBrC and those in WSBrC. We hypothesize that the dominant light-absorbing species in WIBrC are generated along the soot-formation (or BC-formation) pathway^78^ and become more strongly absorbing as they approach the BC-formation threshold. Soot-formation chemistry, which involves growth and clustering of PAHs by radical-chain reactions,^93,94^ is similar across fuel types (including biomass and fossil fuels), thus the light-absorption properties of these species are expected to be highly dependent on combustion conditions (FRE_norm_). This result is in agreement with the report by Chakrabarty et al.^25^ that *k*_550_ of dark BrC in wildfire plumes decreased with decreasing flame temperature. Conversely, the dominant light-absorbing species in WSBrC are polar compounds that are likely specific to biomass burning, such as lignin-pyrolysis and distillation products.^83^ The formation of these species is possibly not strongly dependent on combustion conditions for the range of combustion conditions encountered in wildland fires. Further confirmation of this hypothesis requires detailed chemical speciation that resolves the molecular structure of the major chromophores in WSBrC and WIBrC.

Importantly, the results shown in Figure 6a and 6d suggest that the variables encountered in wildland fires, such as those investigated in this study (fuel-bed composition and moisture content), affect BrC light-absorption properties to the extent that they influence combustion conditions. For surface fires (i.e., fires that consume surface fuels only), combustion conditions are modulated by moisture content. Therefore, capturing the natural variability of light-absorption properties of BrC emissions from surface fires can be efficiently achieved by performing experiments that vary the moisture content of the fuel bed rather than its composition. However, the combustion conditions of ground fires (i.e., fires that consume duff in addition to surface fuels) are modulated by duff ignition. Therefore, to accurately represent BrC emissions from ground fires, it is essential to include duff in the fuel bed.

Correlating BrC light-absorption properties with combustion conditions, specifically FRE_norm_, allows for translating experimental results to modeling platforms. FRE can be derived from satellite observations.^90,91,95^ Fuel mass loading data, typically obtained from satellite observations or field measurements,^96^ is available in emission inventories.^97^ Furthermore, promising techniques to obtain more detailed estimates of wildland fuel loadings, such as LIDAR, have been continually developed,^98−101^ which will lead to more accurate retrievals of FRE_norm_ for various wildland covers. Therefore, FRE_norm_ is a practical basis for parametrizing *k*_550_ and w of wildland-fire BrC in chemical-transport and climate models, allowing for improved representation of the role of wildland-fire aerosol in climate-fire feedback.^102^

## Supporting Information Available

Uncertainty analysis and tables which provide complete data for fuel bed information, OCEC analyzer protocols, and aerosol (BrC, WSBrC, and WIBrC) optical properties (PDF)
Mass spectrometry data including all assigned formulas, the corresponding abundance, mass, and error (ppm) for each analyzed burn (XLSX)
