# Supplementary material for: Brown Carbon Emissions from Biomass Burning under Simulated Wildfire and Prescribed-Fire Conditions
Source: ACS EST Air. 2024 Aug 21;1(9):1124–36. doi: 10.1021/acsestair.4c00089 (PMC11406530; doi:10.1021/acsestair.4c00089)
Supplement: Supplementary file 1 — ea4c00089_si_001.pdf [file ea4c00089_si_001.pdf]

Supporting Information For:

Brown Carbon Emissions from Biomass Burning Under Simulated Wildfire and Prescribed-fire Conditions

*Chase K. Glenn[a‡], Omar El Hajj[a†], Zachary McQueen[b], Ryan P. Poland[b], Robert Penland[a], Elijah T. Roberts[b], Jonathan H. Choi[b], Bin Bai[c], Nara Shin[c], Anita Anosike[a], Kruthika V. Kumar[a], Muhammad Isa Abdurrahman[a], Pengfei Liu[c], I. Jonathan Amster[b], Geoffrey D. Smith[b], Steven Flanagan[d], Mac A. Callaham[d], Eva L. Loudermilk[d], Joseph J. O'Brien[d], Rawad Saleh[a]**

[a] School of Environmental, Civil, Agricultural and Mechanical Engineering, University of Georgia, Athens, Georgia 30602, United States

[b] Department of Chemistry, University of Georgia, Athens, Georgia 30602, United States

[c] School of Earth and Atmospheric Sciences, Georgia Institute of Technology, Atlanta, Georgia 30332, United States

[d] USDA Forest Service Southern Research Station, Athens, Georgia 30602, United States

[‡]Now at Aerodyne Research Inc., Billerica, Massachusetts 01821, United States

[†]Now at Tofwerk USA, Boulder, Colorado 80301, United States

**To whom correspondence should be addressed.* rawad@uga.edu | *(706) 542-6110*

S1. Uncertainty Analysis:

Notes:

S1.1. Uncertainty in TM, the mass of organic and elemental carbon on the quartz filter.

The total mass (TM) mass collected on the quartz filters is calculated from the OCEC measurements as:

$$TM = TM_Q - OC_{QBT} \tag{S1}$$

Where, TM_Q is the OC mass and EC mass on the bare quartz filter and OC_{QBT} is the OC mass on the quartz filter behind the Teflon filter.
The uncertainty in OC is determined based on the uncertainty (sigma) reported by the OCEC as:

$$\sigma_{TM}^2 = \sigma_{TM_Q}^2 + \sigma_{OC_{QBT}}^2 \tag{S2}$$

Where the OCEC reported uncertainty is 5% of the measurement and where σ_{TM_q}, and $\sigma_{OC_{QBT}}$are reported by the OCEC.

S1.2. Uncertainty in OC_{WI} and OC_{WS}, the mass of water-insoluble and water-soluble organic carbon on the quartz filter.

The mass of WSBrC and WIBrC collected on the extracted and unextracted quartz filters calculated as:

$$OC_{WI} = OC_{Q,WI} - OC_{QBT,WI} \tag{S3}$$

$$OC_{QBT,WS} = OC_{QBT} - OC_{QBT,WI} \tag{S4}$$

$$OC_{Q,WS} = OC_Q - OC_{Q,WI} \tag{S5}$$

$$OC_{WS} = OC_{Q,WS} - OC_{QBT,WS} \tag{S6}$$

$$\sigma_{OC_{WI}}^2 = \sigma_{OC_{Q,WI}}^2 + \sigma_{OC_{QBT,WI}}^2 \tag{S7}$$

$$\sigma_{OC_{WS}}^2 = \sigma_{OC_Q}^2 + \sigma_{OC_{QBT}}^2 + \sigma_{OC_{Q,WI}}^2 + \sigma_{OC_{QBT,WI}}^2 \tag{S8}$$

Where σ_{OC_Q}, $\sigma_{OC_{QBT}}$, $\sigma_{OC_{Q,WI}}$, $\sigma_{OC_{QBT,WI}}$ and are obtained from the OCEC analyzer.

S1.3. Uncertainties in the mass fractions of WSBrC, WIBrC, and EC.

Fraction of WSBrC, WIBrC and EC relative to TM, Where OM_{WS} and OM_{WI} is converted to organic matter basis from OC_{WS} and OC_{WI} assuming OM/OC =1.8, and calculated as:

$$f_{WSBrC} = \frac{OM_{WS}}{TM};\ f_{WIBrC} = \frac{OM_{MI}}{TM};\ f_{EC} = \frac{EC}{TM} \tag{S9}$$

$$\sigma^2_{f_{WSBrC}} = \sigma^2_{OM_{WS}}\left(1/_{TM}\right)^2 + \sigma^2_{TM}\left(OM_{WS}/_{TM^2}\right)^2 \tag{S10}$$

$$\sigma^2_{f_{WIBrC}} = \sigma^2_{OM_{WI}}\left(1/_{TM}\right)^2 + \sigma^2_{TM}\left(OM_{WI}/_{TM^2}\right)^2 \tag{S11}$$

$$\sigma^2_{f_{EC}} = \sigma^2_{EC}\left(1/_{TM}\right)^2 + \sigma^2_{TM}\left(EC/_{TM^2}\right)^2 \tag{S12}$$

Where $\sigma_{OM_{WS}}$, $\sigma_{OM_{WI}}$, σ_{TM} are calculated above and σ_{EC} is reported by the OCEC.

S1.4. Uncertainty in k_{WSBrC}

The imaginary part of the refractive index (k) for WSBrC calculated as:

$$k_{WSBrC,\lambda} = \frac{A(\lambda)}{C_{WSBrC}} \times \frac{\ln 10 \rho \lambda}{4\pi L} \tag{S13}$$

$$C_{WSBrC} = C_{Q,WS,extract} - C_{QBT,WS,extract} \tag{S14}$$

$$\sigma^2_{C_{WSBrC}} = \sigma^2_{C_{Q,WS,extract}} + \sigma^2_{OC_{QBT,WS,extract}} \tag{S15}$$

$$\sigma^2_{k_{WSBrC,\lambda}} = \left(\frac{\ln 10 \rho \lambda}{4\pi L}\right)^2 \times \left(\sigma^2_{A(\lambda)}\left(1/_{C_{WSBrC}}\right)^2 + \sigma^2_{C_{WSBrC}}\left(A(\lambda)/_{{C_{WSBrC}}^2}\right)^2\right) \tag{S16}$$

Where $C_{Q,WS,extract}$ and $C_{QBT,WS,extract}$ are the concentrations of the WSBrC solution and $\sigma_{C_{Q,WS,extract}}$ and $\sigma_{C_{QBT,WS,extract}}$ are obtained from the OCEC analyzer. $\sigma_{A(\lambda)}$is 1% of $A(\lambda)$, per manufacturer's specifications.

S1.5. Uncertainty in w

Wavelength dependence (w) used for WSBrC and WIBrC calculated as:

$$w = \frac{\log(k_{406}/k_{532})}{\log(532/406)} \tag{S17}$$

$$\sigma_w^2 = \left(\frac{1}{\ln\left({}^{532}/_{406}\right)}\right)^2 \times \left(\sigma_{k_{406}}^2\left({}^{1}/_{k_{406}}\right)^2 + \sigma_{k_{532}}^2\left({}^{1}/_{k_{532}}\right)^2\right) \tag{S18}$$

For BrC aerosol, $\sigma_{k_\lambda}$is the standard deviation of the averaged k_λ values obtained from Mie theory calculations over the sampling period, in this case 120 seconds. For WSBrC and WIBrC, σ_{k_λ}calculations are described in S1.4 and S1.7, respectively.

S1.6. Uncertainty in k_{550}

k_{550} obtained for BrC aerosol from optical closure and Mie theory calculated as:

$$k_{550} = k_{532}\left({}^{550}/_{532}\right)^w \tag{S19}$$

$$\sigma_{k_{550}}^2 = \sigma_{k_{532}}^2\left({}^{550}/_{532}\right)^w + \sigma_w^2\left(k_{532} \times \ln\left({}^{550}/_{532}\right) \times \left({}^{550}/_{532}\right)^w\right)^2 \tag{S20}$$

Where σ_{k_λ} is obtained identical to S1.4.

S1.7. Uncertainty in k_{WIBrC}

$k_{BrC,aerosol,\lambda}$ is assumed to be the volume weighted average of WSBrC and WIBrC, where $k_{WIBrC,\lambda}$ is calculated as:

$$k_{WIBrC,\lambda} = \left(k_{BrC,aerosol,\lambda} - k_{WSBrC,\lambda}\frac{f_{WSBrC}}{f_{WSBrC} + f_{WIBrC}}\right)\frac{f_{WSBrC} + f_{WIBrC}}{f_{WIBrC}} \tag{S21}$$

$$\begin{aligned}\sigma_{k_{WIBrC,\lambda}}^2 = {} & \sigma_{k_{BrC,aersosl,\lambda}}^2\left(\frac{f_{WSBrC} + f_{WIBrC}}{f_{WIBrC}}\right)^2 + \sigma_{k_{WSBrC,\lambda}}^2\left(\frac{f_{WSBrC}}{f_{WIBrC}}\right)^2 \\ & + \sigma_{f_{WSBrC}}^2\left(\frac{k_{BrC,aerosol,\lambda} - k_{WSBrC,\lambda}}{f_{WIBrC}}\right)^2 \\ & + \sigma_{f_{WIBrC}}^2\left(\frac{k_{BrC,aerosol,\lambda} \times f_{WSBrC} - k_{WSBrC,\lambda} \times f_{WSBrC}}{f_{WIBrC}{}^2}\right)^2\end{aligned} \tag{S22}$$

Where $\sigma_{k_{BrC,aersosl,\lambda}}$ is the standard deviation of the k_λ values calculated from Mie theory calculations, as in S1.4 and where $\sigma_{f_{WSBrC}}$, $\sigma_{f_{WIBrC}}$, and $\sigma_{k_{WSBrC,\lambda}}$ are also previously calculated above.

S1.8. Uncertainty in $X_{abs,EC}$, EC contribution to absorption.

The contribution EC makes to absorption ($b_{abs,EC}$) a fraction of total aerosol absorption (b_{abs}) calculated as:

$$X_{abs,EC} = \frac{b_{abs,EC}}{b_{abs}} \tag{S23}$$

$$\sigma^2_{X_{abs,EC}} = \sigma^2_{b_{abs,EC}} \left(1/_{b_{abs}}\right)^2 + \sigma^2_{b_{abs}} \left(b_{abs,EC}/_{{b_{abs}}^2}\right)^2 \tag{S24}$$

Where $\sigma_{b_{abs}}$ is the standard deviation of the absorption coefficients (b_{abs}) measured using the PAS. with one average $\sigma_{b_{abs}}$ calculated for every 120 seconds of measurement, the length of an SMPS scan. $\sigma_{b_{abs,EC}}$ is the standard deviation of the absorption attributed to EC and is calculated in the same fashion.

S1.9. Uncertainty in $X_{abs,WSBrC}$, WSBrC contribution to absorption.

The fraction of total absorption contributed by WSBrC, where the fractional absorption is assumed to be the weighted average of WSBrC and WIBrC in relation to $k_{BrC,aerosol}$, and where $X_{abs,WSBrC}$ is calculated as:

$$X_{abs,WSBrC} = \left(1 - X_{abs,EC}\right) \frac{\left(k_{WSBrC} \times f_{WSBrC}/(f_{WSBrC} + f_{WIBrC})\right)}{k_{BrC,aerosol}} \tag{S25}$$

$$\begin{aligned}\sigma^2_{X_{abs,WSBrC}} &= \sigma^2_{X_{abs,EC}} \left(\frac{\left(k_{WSBrC} \times f_{WSBrC}/(f_{WSBrC} + f_{WIBrC})\right)}{k_{BrC,aerosol}}\right)^2 \\ &+ \sigma^2_{k_{WSBrC}} \left(\frac{\left(1 - X_{abs,EC}\right) f_{WSBrC}/(f_{WSBrC} + f_{WIBrC})}{k_{BrC,aerosol}}\right)^2 \\ &+ \sigma^2_{f_{WSBrC}} \left(\frac{\left(1 - X_{abs,EC}\right) k_{WSBrC}}{k_{BrC,aerosol}} \times \frac{f_{WIBrC}}{(f_{WSBrC} + f_{WIBrC})^2}\right)^2 \\ &+ \sigma^2_{f_{WIBrC}} \left(\frac{\left(1 - X_{abs,EC,\lambda}\right) k_{WSBrC,\lambda} \times f_{WSBrC}}{k_{BrC,aerosol} \times \ (f_{WSBrC} + f_{WIBrC})^2}\right)^2 \\ &+ \sigma^2_{k_{BrC,aerosol}} \left(\left(1 - X_{abs,EC}\right) \frac{\left(k_{WSBrC} \times f_{WSBrC}/(f_{WSBrC} + f_{WIBrC})\right)}{{k_{BrC,aerosol}}^2}\right)^2\end{aligned} \tag{S26}$$

Where $\sigma_{k_{BrC,aersosl}}$, $\sigma_{f_{WSBrC}}$, $\sigma_{f_{WIBrC}}$, $\sigma_{k_{WSBrC}}$, and $\sigma_{X_{abs,EC}}$ are calculated previously.

S1.10. Uncertainty in $X_{abs,WIBrC}$, WIBrC contribution to absorption.

The fraction of total absorption contributed by WIBrC, where the fractional absorption is assumed to be the weighted average of WSBrC and WIBrC in relation to $k_{BrC,aerosol}$, and where $X_{abs,WIBrC}$ is calculated as:

$$X_{abs,WIBrC} = \left(1 - X_{abs,EC}\right)\frac{\left(k_{WIBrC} \times f_{WIBrC}/(f_{WSBrC} + f_{WIBrC})\right)}{k_{BrC,aerosol}} \tag{S27}$$

$$\begin{aligned}
\sigma^2_{X_{abs,WIBrC}} = \sigma^2_{X_{abs,EC}} &\left(\frac{\left(k_{WIBrC,\lambda} \times f_{WIBrC}/(f_{WSBrC} + f_{WIBrC})\right)}{k_{BrC,aerosol}}\right)^2 \\
+ \sigma^2_{k_{WIBrC,\lambda}} &\left(\frac{\left(1 - X_{abs,EC,\lambda}\right)f_{WIBrC}/(f_{WSBrC} + f_{WIBrC})}{k_{BrC,aerosol}}\right)^2 \\
+ \sigma^2_{f_{WIBrC}} &\left(\frac{\left(1 - X_{abs,EC}\right)k_{WIBrC}}{k_{BrC,aerosol}} \times \frac{f_{WIBrC}}{(f_{WSBrC} + f_{WIBrC})^2}\right)^2 \\
+ \sigma^2_{f_{WSBrC}} &\left(\frac{\left(1 - X_{abs,EC}\right)k_{WIBrC} \times f_{WIBrC}}{k_{BrC,aerosol} \times \ (f_{WSBrC} + f_{WIBrC})^2}\right)^2 \\
+ \sigma^2_{k_{BrC,aerosol}} &\left(\left(1 - X_{abs,EC}\right)\frac{\left(k_{WIBrC} \times f_{WIBrC}/(f_{WSBrC} + f_{WIBrC})\right)}{{k_{BrC,aerosol}}^2}\right)^2
\end{aligned} \tag{S28}$$

Where $\sigma_{k_{BrC,aersosl}}$, $\sigma_{f_{WSBrC}}$, $\sigma_{f_{WIBrC}}$, $\sigma_{k_{WSBrC}}$, and $\sigma_{X_{abs,EC}}$ are calculated previously.

S.2. Extraction efficiency sensitivity test

As described in Section 2.5 of the main text, WSBrC and WIBrC fractions were determined by passively extracting a Q punch in 3 mL of water for 24 hours. To ensure that the apportionment into WSBrC and WIBrC is not sensitive to the operational conditions, we performed experiments to test the sensitivity of the extraction procedure to a) volume of water and b) extraction time.

We generated smoke in the laboratory by burning pine needles inside a 7 m^3 Teflon chamber. We collected two Q filter samples (A and B). The TOC collected on each filter had a concentration of approximately 30 $\mu g/cm^2$, which is within the range of values obtained during G-WISE. Four punches were taken from each filter. One punch was analyzed in the OCEC immediately and the other three punches were passively extracted in ultrapure water for different time points (24 hours, 42 hours, and 48 hours). The extraction volumes were 3 mL and 6 mL for the punches taken from filter A and filter B, respectively. We calculated the fraction of WIBrC

following the same procedure in Section 2.5 in the main text, with the exception that no QBT correction was applied in these experiments.

As shown in Figure S1, the WIBrC fractions exhibited minimal sensitivity to extraction volume and extraction time. This indicates that under our experimental conditions, there were neither kinetic limitations nor solubility limitations associated with the extraction process. Therefore, the WSBrC and WIBrC fractions reported in the main text, though operationally defined, can be practically generalized.

Figures

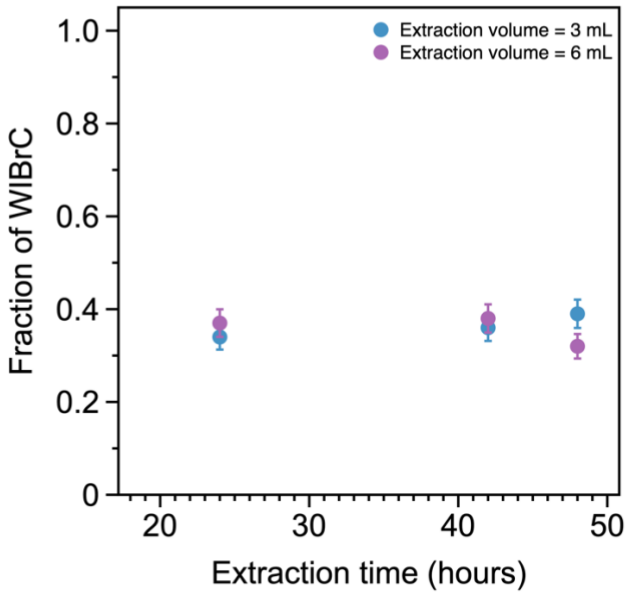

Figure S1. Results of the extraction efficiency test in Section S2.

Tables

Table S1. Dry mass loading and moisture content for wildfire and prescribed-fire conditions of the fuel beds employed during the Georgia Wildland-fire Simulation Experiment (G-WISE).

Ecoregion	Total Mass Loading (g/m^2)	Woody Fuels (1hr, 10hr, 100hr) (g)	Pine Needles (g)	Everything Else *(g)	Duff (g)	Moisture Content (Wild) ** (%)	Moisture Content (Prescribed) ** (%)
Piedmont	500	90	250	160	N/A	Woody: 1%<br>Fine: 1.8% - 2.7%	Woody: 32% - 50%<br>Fine: 10% - 11%
Coastal Plain	500	101	277	122	N/A	Woody: <1%<br>Fine: 2.3% - 2.4%	Woody: 32% - 50%<br>Fine: 10% - 11%
Blue Ridge	2420 - 3240	100	N/A	100	2177-2997	Woody : 1% - 4%<br>Fine: 2.4%<br>Duff: 2.4% - 2.9%	Woody : 32% - 50%<br>Fine: 10%<br>Duff: 50%

* Everything Else refers to fine fuels other than pine needles, including leaves, grasses, etc.
**The category "Fine" includes moisture content range for "Pine Needles" and "Everything Else".

Table S2. NIOSH-870 Protocol for thermal-optical analysis[51].

Carrier gas	Temperature (°C)	Residence time (s)	Carbon Fraction
Helium	310	80	OC1
	475	60	OC2
	615	60	OC3
	870	90	OC4
Oxygen (2%) in helium	550	45	EC1
	625	45	EC2
	700	45	EC3
	775	45	EC4
	850	45	EC5
	870	45	EC6

Table S3. Fractions of organic carbon (OC) bins and elemental carbon (EC) obtained from OCEC analyzer. OC values are corrected for adsorbed vapors as described in Section 2.3 in the main text.

Date	Experiment	OC1	OC2	OC3	OC4	EC
10/25/22	P-Wild	58.8%	19.2%	8.2%	3.6%	10.3%
11/1/22	P-Wild	53.1%	21.6%	13.5%	5.3%	6.5%
10/31/22	P-Rx	50.3%	25.4%	16.0%	4.7%	3.7%
11/2/22	CP-Wild	50.3%	20.7%	14.1%	5.0%	9.8%
11/4/22	CP-Wild	43.6%	21.0%	15.9%	4.7%	14.7%
11/3/22	CP-Rx	59.1%	19.5%	11.5%	4.5%	5.4%
11/6/22	CP-RX	50.7%	22.7%	16.0%	6.1%	4.4%
11/12/22	BR-Wild	65.0%	18.9%	10.8%	3.6%	1.7%
11/14/22	BR-Wild	68.6%	17.1%	9.7%	3.1%	1.4%
11/11/22	BR-Rx	42.8%	28.5%	17.6%	5.8%	5.3%
11/15/22	BR-Rx	45.9%	26.8%	15.8%	6.6%	4.9%

Table S4. Retrieved imaginary part of the refractive index at 550 nm (k_{550}) and its wavelength dependence (w) of BrC aerosol, water-soluble BrC (WSBrC), and water-insoluble BrC (WIBrC). Experiments refer to a combination of ecoregion (Piedmont: P; Coastal Plain: CP; Blue Ridge: BR) and burn condition (Wildfire: Wild; Prescribed fire: Rx).

Date	Experiment	BrC Aerosol		WSBrC		WIBrC	
		k550	w	k550	w	k_{550}	w
10/25/22	P-Wild	0.024 ± 0.00002	1.44 ± 0.02	0.0014 ± 0.00007	6.7 ± 0.26	0.0567 ± 0.003	0.649 ± 0.21
10/27/22	P-Wild	0.02 ± 0.0004	1.26 ± 0.1	—	—	—	—
11/1/22	P-Wild	0.02 ± 0.0006	1.3 ± 0.13	0.0011 ± 0.00006	6.41 ± 0.29	0.0674 ± 0.004	0.4986 ± 0.31
10/28/22	P-Rx	0.009 ± 0.0002	2.87 ± 0.1	—	—	—	—
10/31/22	P-Rx	0.011 ± 0.00001	2.92 ± 0.03	0.0024 ± 0.00016	5.81 ± 0.35	0.0311 ± 0.002	1.1516 ± 0.26
11/2/22	CP-Wild	0.026 ± 0.0006	0.9 ± 0.13	0.0009 ± 0.00005	6.83 ± 0.29	0.0957 ± 0.006	0.3843 ± 0.32
11/4/22	CP-Wild	0.042 ± 0.0012	0.72 ± 0.15	0.0023 ± 0.00017	4.82 ± 0.39	0.1677 ± 0.013	0.3465 ± 0.39
11/8/22	CP-Wild	0.035 ± 0.0008	0.88 ± 0.12	—	—	—	—
11/3/22	CP-Rx	0.01 ± 0.00004	2.15 ± 0.09	0.0012 ± 0.00007	6.38 ± 0.3	0.0414 ± 0.002	0.9866 ± 0.28
11/6/22	CP-RX	0.011 ± 0.0004	2.5 ± 0.19	0.0018 ± 0.00014	5.6 ± 0.39	0.043 ± 0.003	1.5853 ± 0.37
11/7/22	CP-RX	0.01 ± 0.0002	2.52 ± 0.11	—	—	—	—
11/9/22	BR-Wild	0.003 ± 0.0001	3.81 ± 0.17	—	—	—	—
11/12/22	BR-Wild	0.005 ± 0.0003	3.07 ± 0.24	0.0008 ± 0.00003	7.79 ± 0.21	0.0111 ± 0.0008	1.8693 ± 0.29
11/14/22	BR-Wild	0.004 ± 0.0001	3.45 ± 0.16	0.0009 ± 0.00003	7.86 ± 0.2	0.008 ± 0.0004	1.7742 ± 0.19
11/10/22	BR-Rx	0.013 ± 0.0008	2.4 ± 0.27	—	—	—	—
11/11/22	BR-Rx	0.011 ± 0.0017	2.76 ± 0.75	0.002 ± 0.00012	5.44 ± 0.31	0.0243 ± 0.02	2.209 ± 0.76
11/15/22	BR-Rx	0.01 ± 0.0002	2.33 ± 0.09	0.0019 ± 0.00012	5.59 ± 0.33	0.0223 ± 0.02	1.43 ± 0.27

Table S5. Literature values in Figure 3 in the main text. Most of the studies did not report k_{550} and w values directly. Reported properties were converted to k_{550} and w as following the same procedures detailed in Saleh[15].

[a] Upper and lower bounds for all data, as reported by each study

[b] Calculated from the reported ratio (k_{WSBrC}/k_{dBrC}) in Chakrabarty et al.[25]

[c] Calculated as the average of all data points reported by Chakrabarty et al.[25]

Ref.	Reported properties	k_{550}	w
**Laboratory: Smoldering (Negligible BC)**			
McClure et al.[34]	k_{405} and w	9×10^{-4}	7.6
Browne et al.[27]	k_{532} and AAE	8.1×10^{-4}	6.2
Sumlin et al.[28]	k vs λ	0.0026	4.4
**Laboratory: BC Producing**			
Atwi et al.[29]	k_{550} and w	0.023 ± 0.01	2.6 ± 0.9
McClure et al.[34]	k_{405} and w	0.038	1.9
McClure et al.[34]	k_{405} and w	0.015	2.5
McClure et al.[34]	k_{405} and w	0.007	3.1
McClure et al.[34]	k_{405} and w	0.0063	3.4
McClure et al.[34]	k_{405} and w	0.004	4.5
Saleh et al.[30]	k_{550} and w	0.01	3.1
Saleh et al.[30]	k_{550} and w	0.024	1.5
Saleh et al.[30]	k_{550} and w	0.011	2.1
Saleh et al.[30]	k_{550} and w	0.028	1.4
Saleh et al.[30]	k_{550} and w	0.07	1.4
Saleh et al.[30]	k_{550} and w	0.012	1.9
Saleh et al.[30]	k_{550} and w	0.03	0.6
**Wildfire Plume**			
[a]Zeng et al.[24]	MAC_{532} and AAE	0.023 (0.006, 0.053)	2.1 (0.26, 3.7)
**Laboratory: WSBrC/MSBrC**			
[a]Atwi et al.[29]	k_{550} and w	0.0032 (0.0022, 0.0042)	6.3 (4.6, 8)
Cheng et al.[32]	k_{405} and w	0.0022	5.9
Li et al.[31]	k vs λ	0.0048	6.2
**Wildfire Plume: WSBrC/MSBrC**			
[b]Chakrabarty et al.[25]	k_{WSBrC}/k_{dBrC}	0.003	6.2
[a]Zeng et al.[24]	MAC_{532} and AAE	0.002 (0.0012, 0.0033)	3.47 (2.16, 4.67)
**Laboratory: Strongly Absorbing (Dark) BrC**			
[a]Atwi et al.[29]	k_{550} and w	0.2 (0.1, 0.3)	1.7 (0.6, 2.8)
Adler et al.[33]	MAC vs λ	0.21	1.5
Saleh et al.[30]	k_{550} and w	0.22	1.1
Saleh et al.[30]	k_{550} and w	0.15	1.4
Saleh et al.[30]	k_{550} and w	0.12	2
**Wildfire Plume: Strongly Absorbing (Dark) BrC**			
[c]Chakrabarty et al.[25]	k vs λ	0.079	0.97

Table S6. Mass fractions and fractional contribution to absorption at 406 nm, 532nm, and 660 nm of water-soluble BrC (WSBrC), water-insoluble BrC (WIBrC), and elemental carbon (EC). Experiments refer to a combination of ecoregion (Piedmont: P; Coastal Plain: CP; Blue Ridge: BR) and burn condition (Wildfire: Wild; Prescribed fire: Rx).

Date	Experiment	WSBrC				WIBrC				EC			
		WS/TM	406 nm	532 nm	660 nm	WI/TM	406 nm	532 nm	660 nm	EC/TM	406 nm	532 nm	660 nm
10/25/22	P-Wild	56% ± 3.4%	15.9% ± 0.9%	3.2% ± 0.1%	0.5% ± 0.2%	38.6% ± 1.6%	71.6% ± 3.9%	75.8% ± 4.9%	65.7% ± 2.7%	5.4% ± 0.3%	12.4% ± 0.1%	20.8% ± 0.2%	33.8% ± 0.4%
11/1/22	P-Wild	69% ± 4.0%	17.4% ± 1.0%	3.8% ± 0.2%	0.6% ± 0.2%	27.6% ± 1.3%	73.2% ± 5.4%	79.6% ± 7.0%	76.0% ± 3.5%	3.2% ± 0.3%	9.3% ± 0.1%	16.5% ± 0.2%	23.5% ± 0.5%
10/31/22	P-Rx	69% ± 4.6%	40.3% ± 2.9%	15.0% ± 1.0%	4.9% ± 1.4%	29.3% ± 1.5%	54.3% ± 3.5%	71.5% ± 5.8%	64.7% ± 3.7%	1.8% ± 0.2%	5.3% ± 0.1%	13.3% ± 0.1%	30.4% ± 0.4%
11/2/22	CP-Wild	70% ± 4.3%	13.9% ± 0.8%	2.5% ± 0.1%	0.1% ± 0.1%	25.5% ± 1.2%	74.2% ± 5.9%	78.7% ± 6.9%	74.7% ± 4.3%	4.9% ± 0.4%	11.8% ± 0.2%	18.6% ± 0.3%	25.3% ± 0.6%
11/4/22	CP-Wild	70% ± 4.7%	11.7% ± 0.9%	3.6% ± 0.3%	2.1% ± 0.3%	22.3% ± 1.3%	74.8% ± 7.1%	77.1% ± 8.0%	72.2% ± 4.0%	7.6% ± 0.6%	13.4% ± 0.3%	19.2% ± 0.5%	25.7% ± 0.8%
11/3/22	CP-Rx	76% ± 4.1%	31.5% ± 2.0%	8.6% ± 0.5%	0.6% ± 0.6%	21.2% ± 1.1%	58.0% ± 4.1%	68.7% ± 5.7%	65.2% ± 3.1%	2.7% ± 0.3%	10.3% ± 0.2%	22.5% ± 0.6%	34.2% ± 1.0%
11/6/22	CP-RX	76% ± 4.8%	29.7% ± 2.5%	10.9% ± 0.9%	4.5% ± 1.1%	22.1% ± 1.2%	60.0% ± 5.4%	65.1% ± 7.6%	59.9% ± 3.7%	2.1% ± 0.2%	10.1% ± 0.2%	23.8% ± 0.3%	35.6% ± 1.6%
11/12/22	BR-Wild	55% ± 3.6%	33.1% ± 2.2%	8.4% ± 0.5%	2.0% ± 0.5%	43.6% ± 1.8%	62.5% ± 4.5%	79.2% ± 7.8%	74.8% ± 3.8%	0.9% ± 0.1%	4.3% ± 0.1%	12.2% ± 0.4%	23.2% ± 1.0%
11/14/22	BR-Wild	56% ± 3.6%	43.4% ± 2.7%	12.0% ± 0.6%	4.1% ± 1.1%	43.3% ± 1.8%	52.8% ± 3.1%	75.9% ± 5.5%	73.4% ± 5.0%	0.7% ± 0.1%	3.6% ± 0.1%	11.9% ± 0.3%	22.6% ± 1.2%
11/11/22	BR-Rx	58% ± 4.7%	34.5% ± 3.4%	14.6% ± 1.5%	4.7% ± 0.9%	39.1% ± 2.1%	55.8% ± 12.6%	63.2% ± 19.3%	55.9% ± 4.7%	2.8% ± 0.4%	9.5% ± 0.5%	22.1% ± 1.2%	39.4% ± 2.5%
11/15/22	BR-Rx	59% ± 4.5%	28.3% ± 2.1%	10.0% ± 0.7%	4.1% ± 0.8%	38.3% ± 2.1%	60.7% ± 4.2%	66.5% ± 6.1%	57.9% ± 3.4%	2.7% ± 0.4%	10.8% ± 0.3%	23.3% ± 0.6%	38.0% ± 1.2%

Table S7. Normalized fire radiative energy. Experiments refer to a combination of ecoregion (Piedmont: P; Coastal Plain: CP; Blue Ridge: BR) and burn condition (Wildfire: Wild; Prescribed fire: Rx).

Date	Experiment	FRE (MJ)	FRE_{norm} (MJ/kg)
10/25/22	P-Wild	0.768	1.53
10/27/22	P-Wild	0.797	1.59
11/1/22	P-Wild	0.513	1.03
10/28/22	P-Rx	0.415	0.831
10/31/22	P-Rx	0.399	0.798
11/2/22	CP-Wild	0.757	1.51
11/4/22	CP-Wild	0.697	1.39
11/8/22	CP-Wild	0.617	1.23
11/3/22	CP-Rx	0.388	0.776
11/6/22	CP-RX	0.345	0.689
11/7/22	CP-RX	0.384	0.769
11/9/22	BR-Wild	1.556	0.480
11/12/22	BR-Wild	1.402	0.468
11/14/22	BR-Wild	1.668	0.562
11/10/22	BR-Rx	0.152	0.758
11/11/22	BR-Rx	0.137	0.685
11/15/22	BR-Rx	0.132	0.658
